# Genetic least square estimation approach to wind power curve modelling and wind power prediction

**DOI:** 10.1038/s41598-023-36458-w

**Published:** 2023-06-06

**Authors:** Zhiming Wang, Xuan Wang, Weimin Liu

**Affiliations:** School of Mechanical and Electrical Engineering, Lanzhou University of Technology, Lanzhou, 730050 China

**Keywords:** Energy science and technology, Renewable energy, Wind energy

## Abstract

Wind power curve (WPC) is an important index of wind turbines, and it plays an important role in wind power prediction and condition monitoring of wind turbines. Motivated by model parameter estimation of logistic function in WPC modelling, aimed at the problem of selecting initial value of model parameter estimation and local optimum result, based on the combination of genetic algorithm and least square estimation method, a genetic least square estimation (GLSE) method of parameter estimation is proposed, and the global optimum estimation result can be obtained. Six evaluation indices including the root mean square error, the coefficient of determination *R*^2^, the mean absolute error, the mean absolute percentage error, the improved Akaike information criterion and the Bayesian information criterion are used to select the optimal power curve model in the different candidate models, and avoid the model’s over-fitting. Finally, to predict the annual energy production and output power of wind turbines, a two-component Weibull mixture distribution wind speed model and five-parameter logistic function power curve model are applied in a wind farm of Jiangsu Province, China. The results show that the GLSE approach proposed in this paper is feasible and effective in WPC modelling and wind power prediction, which can improve the accuracy of model parameter estimation, and five-parameter logistic function can be preferred compared with high-order polynomial and four-parameter logistic function when the fitting accuracy is close.

## Introduction

With the social and economic development, the process of urbanization is inseparable from the massive use of energy. The dramatic increase in energy demand has led to substantial consumption of non-renewable resources such as coal and oil. However, the traditional energy reserves are limited and cannot be exploited and utilized without restraint. At the same time, the burning of coal, oil and other fossil energy is seriously harmful to the atmosphere, such as the hazy weather in cities. High emissions of carbon dioxide and other greenhouse gases have caused the global environmental problems. Therefore, the demand for green, clean and renewable energy such as wind energy and solar energy is increasingly anticipated.

In the past decades, wind energy has been developed rapidly, but it has many uncertainties compared with the traditional fossil energy. Therefore, an accurate and effective assessment method of wind energy is of great importance for studying large-scale wind power grid connection and wind farm site selection^1–4^. To estimate wind turbine power, the volatility and intermittency of wind power system is generally investigated by establishing a mathematical model in statistics method. Nevertheless, the process of modelling is complicated because of the stochastic nature, bimodal or multimodal distributions of wind speed^5^. Wind power curve (WPC), which expresses the nonlinear relationship between the hub height wind speed and the actual power output of wind turbines, is commonly used to estimate wind resource in a wind farm^6–13^. Besides wind resource assessment and prediction, WPC is also play an important role in status monitoring of wind turbines. Therefore, WPC is an important performance metric of wind turbines and it is crucial to establish an accurate and reliable WPC^14–17^. For various reasons, raw wind data contain some outliers which caused by the faulty of wind turbines and measurement equipment, and extreme weather, etc. To improve the prediction accuracy of wind power, these abnormal data must be cleaned before WPC modelling^18,19^. In literature, there are two kinds of methods widely used for cleaning abnormal data: (1) clustering method or image recognition method using wind speed-power data and (2) mean value, variance, and probability distribution method based on the distribution characteristics of abnormal data. In this study, a Bayesian change point-quartile combined method is utilized to clean abnormal data^20^.

**Figure 1:**
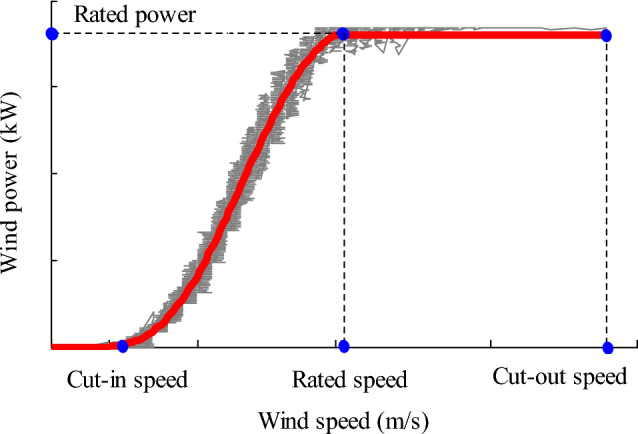
Wind turbine power curve. Figure created using Matlab R2014a (8.3.0.532). (https://www.mathworks.com).

According to the modelling theory of WPC, the WPC modelling methods in literature are divided into two categories: parametric and nonparametric methods^10,21^. Among them, the parametric models are most widely applied, such as a piecewise cubic polynomial model. The advantage of cubic polynomial is that its S-shaped conforms to theoretical power curve of wind turbines. Before modelling, WPC is often divided into three segments by the cut-in, cut-out, and rated wind speed, then a segmented WPC will be obtained using a cubic polynomial fitting technique^2,3^. Figure 1 is a specific wind turbine power curve fitted by a high-order polynomial after cleaning abnormal data.

Wang et al.^4^ compared the performance of various types of power curves at different wind farms and seasons, and pointed out that there exists no universal WPC model which can always outperform other models under any environmental conditions, each model has its own advantages, and three main factors which affect the final WPC are abnormal data cleaning method, WPC model and selection of optimization strategies. Carrillo et al.^1^ compared high-order polynomial with exponential power and cubic polynomial in power curve modelling, and found that high-order polynomial has little improvement on modelling accuracy due to its sensitivity to the values of model parameters, especially to the rated wind speed value. The logistic function (LF), which including three-parameter (3PLF), four-parameter (4PLF) and five-parameter logistic functions (5PLF), is widely applied in WPC modelling because of its S-shaped, continuity and low errors^2,22,23^. LF is also called S-shaped function or sigmoid function, it is originally applied for fitting S-shaped curve in models of population growth and spread of epidemic diseases^35^. In these cases, the growth is exponential with the time at the beginning, then some kind of competition appears among the members of the population, so the growth decreases and finally the size of the population reaches its limit. The shape of WPC happens to meet this condition and there exist some analogy between the population growth and the output power: the wind speed equivalent to the time is also increased gradually to obtain the output power, and at last the output power in a limit corresponding to the rated power, so now LF is widely used in WPC modelling. Villanueva and Feijoo studied LF from 3 to 6PLF and used the mean MAPE as the indicator to compare their performances, they considered that the errors made by the 3PLF are approximately the same as those made by the 4PLF, the 6PLF is the best option to model a WPC. However, dealing with six parameters is cumbersome^23^. Same as Villanueva and Feijoo’s work, zou et al. also studied the LF in WPC modelling, they found that the performances of the 3-PLF and 6-PLF models are slightly inferior to those of other models, regardless of the loss function used^29^. Therefore, in this paper, we study 4PLF and 5PLF models of WPC. However, it is not easy to estimate model parameters for LF, especially for 5PLF, because it has more model parameters. Generally, when using a parametric method to estimate model parameters of LF, an effective initial value is needed because of the nonlinearity of LF. Using the traditional widely used optimization methods such as steepest descent, Levenberg–Marquardt, Newton, and quasi-Newton methods, model parameters can be estimated by an iterative approach. However, these optimization methods are very sensitive to the initial values of the unknown parameters and often fail to converge to the global optimum of the parameter estimation, the quality of the final solution is often dependent upon the position of the initial value in the search space, and there is no guarantee that the procedure can fit the model successfully. Therefore, the initial value is an important factor affecting the convergence of nonlinear model fitting. If the initial value is not selected properly, the final results will fall into a local optimum. In literature, the least square estimate (LSE) method is also used to estimate model parameters of WPC^8^. Using this method, the optimal power curve can be obtained by minimizing the summed square between the predicted and observed power values. However, for a complex model with nonlinear function, the partial derivatives of the function with respect to model parameters are difficult to calculate and estimate^1,24–26^. To solve this problem, an optimization algorithm is often combined, such as whale optimization algorithm (WOA), particle swarm optimization algorithm (PSOA), genetic algorithm (GA), differential evolution algorithm (DEA), and evolutionary algorithm (EA)^2,27–29^. GA is a robust probabilistic search algorithm combining the mechanics of genetic, it searches the optimal solution based on a population instead of a single point. Therefore, when GA is used to estimate model parameter, it is possible to escape from the local optimum and find the global optimum at a certain probability. In the problem of the selection of initial values, GA require an estimate of the parameter range in which the solution values would be found for the problem. This is because that GA has many potential solutions approach and can search multiple points simultaneously. To improve the predictive accuracy of wind power, other factors including air density, wind shear, age of turbines and wind curtailment are also considered in WPC modelling^30–32^.

Compared with parametric method, non-parametric method does not need the assumption about the distribution of data, so it is more flexible than parametric method, but require a lot more data and training time, and cannot give a definite expression to explicitly reflect the relationship between wind speed and power because of its “black box” nature^5,29^. Artificial neural network (ANN) method has an extremely wide range of applications and has been used in WPC modelling, which has the advantages including small error results and simple parameter estimation^25,33^. However, ANN method depends on massive data training to obtain reliable results, and the disadvantages of slow training speed and high data volume requirements are also significant. Fuzzy clustering (FC) method can be used in WPC modelling through finding cluster centers, and can further improve the modelling accuracy by increasing the number of clusters and reducing root mean squared error (RMSE) between the observed values and predicted values, but FC method has a slow convergence speed, and the efficiency and effectiveness of these techniques are highly dependent on the optimal choice of model parameters, so it is often applied in combination with other methods^24^.

**Figure 2:**
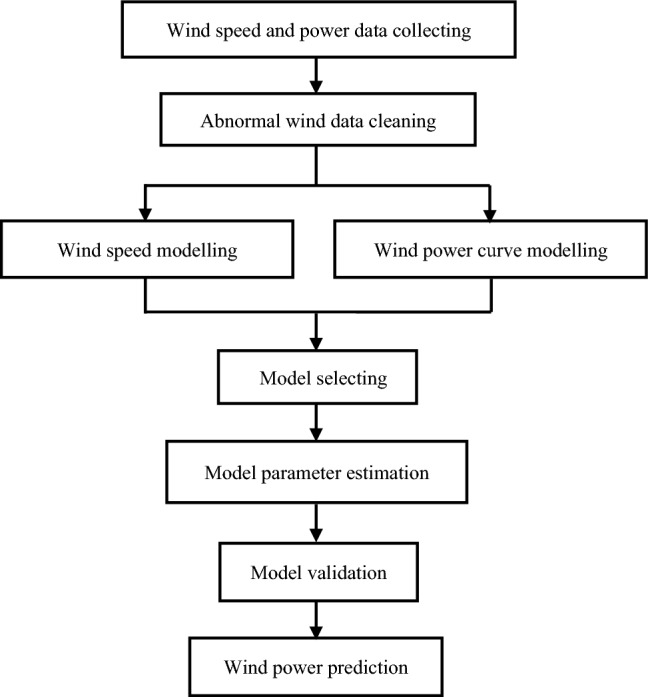
The overall flow chart of the study.

Consequently, in this paper, to model WPC using LF, a global optimization GA algorithm combined with the LSM method named the GLSM method are used to estimate model parameters, which can obtain an effective initial value and ensure the estimation results of logistic model parameter is effective and reliable. The overall flow chart of this study is given in Fig. 2.

The main contributions of this paper are listed below:

(i) Aiming at the problem of the selecting of initial value of model parameter estimation for LF in WPC modelling using the LSE method, a GLSE method is proposed, which can obtain a global optimum estimation results.

(ii) In addition to considering RMSE, the coefficient of determination *R*^2^, mean absolute error (MAE) and mean absolute percentage error (MAPE) are used as a criteria of model selection, the improved Akaike information criterion (AIC) and Bayesian information criterion (BIC) are also utilized to select the best wind power model and avoid the problem of model’s over-fitting.

The rest of this paper is organized as follows. In “Methodology”, WPC model parameter estimation, selection and validation are given. Wind power estimation is described in “Wind speed modelling and wind power prediction”. While in “Case study” some information about the observed field and wind data are provided. Results and comparison with the different models are presented in “Results and discussion”. Conclusions are drawn in “Conclusion”.

## Methodology

To establish a WPC, it is necessary to select fitting points from a large amount of normal data of wind speed and power. These wind data could come from experimental wind farms or from the Supervisory Control and Data Acquisition (SCADA) system^5,34^. At present, the main methods of choice of fitting point for WPC include the bins method, maximum value method, and maximum likelihood method^5^. Among them, the bins method is the most widely used. According to the IEC-61400-12-2 standard, the principle of the bins method is that after eliminating the outliers of wind power data, the average value of wind speed and power in each wind speed interval (the size of the interval is 0.5 m/s) can be got, and these points are used as the sample points for fitting power curve, whose expression is given by^5^

1 $$ v_{i} = \frac{1}{{n_{i} }}\sum\limits_{j = 1}^{{n_{i} }} {v_{i,j} } , \, P_{i} = \frac{1}{{n_{i} }}\sum\limits_{j = 1}^{{n_{i} }} {P_{i,j} } $$

where *v*_*i*_ and *P*_*i*_ are the average wind speed and power in the *i*th interval, *n*_*i*_ is the number of wind data in the *i*th interval, *v*_*i, j*_ and *P*_*i, j*_ are the* j*th wind speed and power in the *i*th interval*.*

### Wind power curve model

As mentioned above, the parametric model of WPC is most widely utilized^10,21^. At present, the commonly used parametric models are high-order polynomial and LF, which describe the relationship between wind speed and power of a specific wind turbine with a mathematical formula. The power expression with an *m*-order polynomial is given by^3,4^:

2 $$ P(v) = a_{0} + a_{1} v + a_{2} v^{2} + \cdots + a_{m} v^{m} = \sum\limits_{i = 0}^{m} {a_{i} } v^{i} $$

where *P*(*v*) is the power value corresponding to wind speed *v*, *m* is the order of polynomial, and ***α*** = [*a*_0_, *a*_1_, …,* a*_*m*_] is the coefficient.

The expressions of 4PLF and 5PLFs are given respectively by^35^

3 $$ P(v) = a\frac{{1 + b\exp \left( {{{ - v} \mathord{\left/ {\vphantom {{ - v} d}} \right. \kern-0pt} d}} \right)}}{{1 + c\exp \left( {{{ - v} \mathord{\left/ {\vphantom {{ - v} d}} \right. \kern-0pt} d}} \right)}} $$

and

4 $$ P(v) = u + \frac{l - u}{{\left[ {1 + \left( {{v \mathord{\left/ {\vphantom {v x}} \right. \kern-0pt} x}} \right)^{y} } \right]^{z} }} $$

where ***θ***_4_ = [*a*,* b*, *c*,* d*] is model parameters that determine the shape of 4PLF, and *a* is the maximum value, the other three parameters have no specific meaning; ***θ***_5_ = [*u*, *l*, *x*, *y*, *z*] is model parameters of 5PLF, *u* and *l* represent the maximum and minimum values, respectively, *x* is the inflection point, *y* is the hill slope, and *z* is the asymmetry factor, with *x* ≥ 0, *z* ≥ 0.

### Model parameter estimation using LSE method

A polynomial is a linear function of unknown model parameters ***α*** = [*a*_0_, *a*_1_, …,* a*_*m*_], thus the techniques of LSE for fitting linear model can be used for fitting polynomial model. Suppose that *n* wind speed and power data pairs (*v*_*i*_, *P*_*i*_) (*i* = 1, 2, …, *n*) are obtained using the bins method, according to Eq. (2), a system of equations of *m*-th order polynomial can be given by

5 $$ \left\{ {\begin{array}{*{20}c} {P_{1} = a_{0} + a_{1} v_{1} + a_{2} v_{1}^{2} + \cdots + a_{m} v_{1}^{m} } \\ {P_{2} = a_{0} + a_{1} v_{2} + a_{2} v_{2}^{2} + \cdots + a_{m} v_{2}^{m} } \\ \cdots \\ {P_{n} = a_{0} + a_{1} v_{n} + a_{2} v_{n}^{2} + \cdots + a_{m} v_{n}^{m} } \\ \end{array} } \right. $$

Rewritten Eq. (5) in matrix by

6 $$ {\varvec{Y}} = \left[ {\begin{array}{*{20}c} {P_{1} } \\ {P_{2} } \\ \vdots \\ {P_{n} } \\ \end{array} } \right]_{n \times 1} , \, {\varvec{X}} = \left[ {\begin{array}{*{20}c} 1 & {v{}_{1}} & {v_{1}^{2} } & \cdots & {v_{1}^{m} } \\ 1 & {v_{2} } & {v_{2}^{2} } & \cdots & {v_{2}^{m} } \\ \vdots & \vdots & \vdots & \ddots & \vdots \\ 1 & {v_{n} } & {v_{n}^{2} } & \cdots & {v_{n}^{m} } \\ \end{array} } \right]_{{n \times \left( {m + 1} \right)}} , \, {\varvec{A}} = \left[ {\begin{array}{*{20}c} {a_{0} } \\ \begin{gathered} a_{1} \hfill \\ a_{2} \hfill \\ \end{gathered} \\ \vdots \\ {a_{m} } \\ \end{array} } \right]_{{\left( {m + 1} \right) \times 1}} $$

Thus

7 $$ {\varvec{Y}} = {\varvec{XA}} $$

Therefore, the model parameters of *m*-th order polynomial can be obtained by^4^

8 $$ {\varvec{A}} = \left( {{\varvec{X}}^{{\text{T}}} {\varvec{X}}} \right)^{ - 1} {\varvec{X}}^{{\text{T}}} {\varvec{Y}} $$

For a LF, due to its highly non-linearity, when using the non-linear least squares estimation (NLLSE) method to fit WPC model, the model parameters can be estimated by an iteration procedure, the aim of iteration is to minimize the summed squares of the residuals between the real values and estimation values, the summed squares of residuals also called objective function defined as

9 $$ e = \min \sum\limits_{i = 1}^{n} {\left[ {P_{i} - P\left( {v_{i} ;{\varvec{\theta}}} \right)} \right]^{2} } $$

where *P*_*i*_ is the real wind power, *P*(*v*_*i*_; ***θ***) is the estimated power, ***θ*** is the vector of unknown model parameters.

NLLSE method is a form of LSE that is used to fit a nonlinear model with *n*_*p*_ unknown model parameters to *n* observations (*n* > *n*_*p*_). Computationally, NLLSE are solved through successive iterations of a two-step process. First, the selected nonlinear mathematical model is approximately linearized around an arbitrary value ***θ***^(*k*)^ of model parameters using a first-order Taylor expansion as follows:

10 $$ P\left( {v_{i} ;{\varvec{\theta}}} \right) \doteq P\left( {v_{i} ;{\varvec{\theta}}^{\left( k \right)} } \right) + \sum\limits_{j = 1}^{{n_{p} }} {\left[ {\frac{{\partial P(v_{i} ;{\varvec{\theta}})}}{{\partial \theta_{j} }}} \right]}_{{{\varvec{\theta}} = {\varvec{\theta}}^{\left( k \right)} }} \left( {\theta_{j} - \theta_{j}^{\left( k \right)} } \right)\;i = 1,2, \cdots ,n; \, j = 1,2, \cdots ,n_{p} ; \, k = 0,1,2, \cdots $$

Secondly, after the estimator of model parameters are solved using the LSE method, the error between the real values and estimated value are calculated. The two steps are repeated till an allowable minimum error is obtained. Note that by taking the first order Taylor expansion of *P*(*v*_*i*_; ***θ***) at an arbitrary point ***θ***^**(***k***)**^ given it is differentiable, then as ***θ*** is close to ***θ***^**(***k***)**^, it gives an approximation. The details of NLLSE method are given as follows:

Step 1. Model approximately linearized Taylor expansion.

Replacing the left-hand side of Eq. (10) with *P*_*i*_ and giving a mathematical transformation, Eq. (11) can be got as

11 $$ P_{i} - P\left( {v_{i} ;{\varvec{\theta}}^{\left( k \right)} } \right) \doteq \sum\limits_{j = 1}^{{n_{p} }} {\left[ {\frac{{\partial P(v_{i} ;{\varvec{\theta}})}}{{\partial \theta_{j} }}} \right]}_{{{\varvec{\theta}} = {\varvec{\theta}}^{\left( k \right)} }} \left( {\theta_{j} - \theta_{j}^{\left( k \right)} } \right) $$

Let $$\Delta P_{i}^{\left( k \right)} = P_{i} - P\left( {v_{i} ;{\varvec{\theta}}^{\left( k \right)} } \right), \, D_{i,j}^{\left( k \right)} = \left[ {\frac{{\partial P(v_{i} ;{\varvec{\theta}})}}{{\partial \theta_{j} }}} \right]_{{{\varvec{\theta}} = {\varvec{\theta}}^{\left( k \right)} }} , \, \Delta \theta_{j}^{\left( k \right)} = \left( {\theta_{j} - \theta_{j}^{\left( k \right)} } \right)$$, then

12 $$ \Delta P_{i}^{\left( k \right)} \doteq D_{i,1}^{\left( k \right)} \Delta \theta_{1}^{\left( k \right)} + D_{i,2}^{\left( k \right)} \Delta \theta_{2}^{\left( k \right)} + \cdots + D_{{i,n_{p} }}^{\left( k \right)} \Delta \theta_{{n_{p} }}^{\left( k \right)} = \sum\limits_{j = 1}^{{n_{p} }} {D_{i,j}^{\left( k \right)} } \Delta \theta_{j}^{\left( k \right)} $$

Equation (12) is a linear combination, rewritten in matrix form by

13 $$ \Delta {\varvec{P}}^{\left( k \right)} \doteq {\varvec{D}}^{\left( k \right)} \Delta {\varvec{\theta}}^{\left( k \right)} $$

Step 2. Parameter estimation and error calculating.

Using the LSE method, Eq. (14) can be obtained as

14 $$ \Delta {\varvec{\theta}}^{\left( k \right)} \doteq \left( {\left( {{\varvec{D}}^{\left( k \right)} } \right)^{T} {\varvec{D}}^{\left( k \right)} } \right)^{ - 1} \left( {{\varvec{D}}^{\left( k \right)} } \right)^{T} \Delta {\varvec{P}}^{\left( k \right)} $$

At last, the (*k* + 1)th approximate estimators of the model parameters are calculated by

15 $$ {\varvec{\theta}}^{{\left( {k + 1} \right)}} = \Delta {\varvec{\theta}}^{\left( k \right)} + {\varvec{\theta}}^{\left( k \right)} $$

The iterative process is stopped when an allowable minimum error is reached.

### Initial value problem of parameter estimation and GLSE method

**Figure 3:**
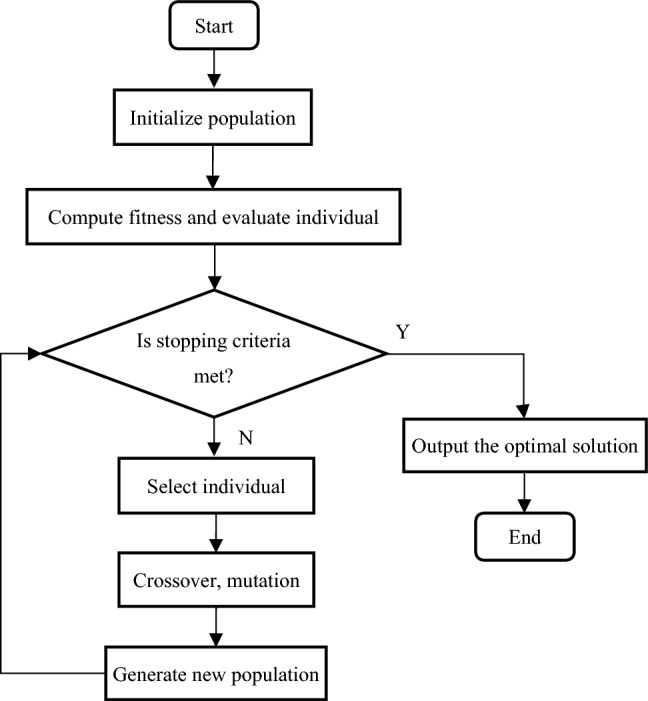
Flow chart of GA approach.

To reduce calculation times and enable iteration convergence, a good initial value ***θ***^**(**0**)**^ should be initiated before the iterative regression process. Unlike a high-order polynomial, using the 4PLF and 5PLFs to fit WPC, the model parameters cannot be estimated directly. Because the choice of initial value, which has a great impact on the final fitting result, is needed. If the initial value is not chosen properly, a local optimum result instead of a global optimum will be obtained. Therefore, before using a LF to fit power curve, it is necessary to find an appropriate initial value by GA. GA is a global search technique based on a combination of natural laws and genetics, including competition, variation and evolution. In contrast to the most optimization methods, GA do not require an initial guess, since they initiate the heuristic solution procedure with a randomly generated population within the solution space. GA also do not require exact or approximate calculations of function derivatives. According to the rule of survival of the fittest, the individuals with higher fitness are more inherited to the next population. The repeat iterations ultimately result in an optimal individual whose phenotype will reach or approach the optimal solution. Therefore, GA is used to solve the initial problem of parameter estimation for LF fitting. The flow chart of GA is shown in Fig. 3.

The parameter set of LF can be regarded as an individual of the population. For a LF with *n*_*p*_ parameters, the individual is represented as a vector of length *n*_*p*_. Suppose that there are *M* individuals in the population, all population is given by a matrix as follows:

16 $$ \left[ {\begin{array}{*{20}c} {{\text{X}}_{11} } & {{\text{X}}_{12} } & \cdots & {{\text{X}}_{1j} } & \cdots & {{\text{X}}_{1M} } \\ {{\text{X}}_{21} } & {{\text{X}}_{22} } & \cdots & {{\text{X}}_{2j} } & \cdots & {{\text{X}}_{2M} } \\ \vdots & \vdots & \vdots & \vdots & \vdots & \vdots \\ {{\text{X}}_{{n_{p} 1}} } & {{\text{X}}_{{n_{p} 2}} } & \cdots & {{\text{X}}_{{n_{p} j}} } & \cdots & {{\text{X}}_{{n_{p} M}} } \\ \end{array} } \right] $$

where ${\text{X}}_{j} = \left[ {{\text{X}}_{1j} ,{\text{X}}_{2j} , \cdots ,{\text{X}}_{{n_{p} j}} } \right]^{T}$ represents the *j*th individual, *X*_*ij*_ is the *j*th estimation solution of the *i*th parameter.

The objection function is defined as the same as Eq. (9). The main processes of GA are given as follows:

*Step* 1 Initialization: The population is initialized randomly within the minimum and maximum limits of the parameters of LF. The constraints of LF are given as ***θ***_*l*_ ≤ ***θ*** ≤ ***θ***_*u*_.

*Step* 2 Evaluation: GA can only handle maximization problems, the fitness value is taken as the inverse of the objective function, therefore, Eq. (17) is selected as the fitness function to calculate and evaluate each individual in the population.

17 $$ f_{GA} = \frac{1}{{\sum\nolimits_{i = 1}^{n} {\left[ {P_{i} - P\left( {v_{i} ;{\varvec{\theta}}} \right)} \right]^{2} } }} $$

*Step* 3 Selection: *M* individuals are selected based on stochastic uniform selection and the fitness values. And the individuals who have better fitness values may have a higher chance of being selected as parents to constitute the new population by crossover and mutation.

*Step* 4 Crossover: The crossover operator combines two parents to produce a child for the next generation. Let parent chromosomes *X*_1_ and *X*_2_ are selected randomly to be crossed, parameter *r* be a random number chosen from [0, 1], and *P*_*c*_ be the crossover probability usually between 0.6 and 0.9. Arithmetic crossover operator is used here. If *r* ≤ *p*_*c*_, then the offspring *Y*_1_ and *Y*_2_ are created as follows^36^:

18 $$ Y_{1i} = r_{i} X_{1i} + \left( {1 - r_{i} } \right)X_{2i} , \, Y_{2i} = r_{i} X_{2i} + \left( {1 - r_{i} } \right)X_{1i} , \, i = 1,2, \cdots ,n_{p} $$

*Step* 5 Mutation: The mutation operator introduces new genetic structures into the population and generate a few random changes in the individuals through the population. It avoids the trap of local minimum and provides generic diversity in the population. The mutation probability *p*_*m*_ is generally between 0.01 and 0.1. Non-uniform mutation operator is applied, then a new mutation offspring would be generated as^36^

19 $$ Y_{i} = \left\{ \begin{gathered} X_{i} + (\theta_{u} - X_{i} )f\left( g \right){\text{ if }}r_{1} < 0.5 \hfill \\ X_{i} - (X_{i} + \theta_{l} )f\left( g \right){\text{ if }}r_{2} \ge 0.5 \hfill \\ X_{i} {\text{ otherwise}} \hfill \\ \end{gathered} \right. $$

where *f*(*g*) = [*r*_2_(1-(*g*/*G*_max_))]^*h*^, *g* is the current generation, *h* is the shape parameter, *G*_max_ is the maximum number of generations.

If the maximum number of iterations is not reached, the above procedure is repeated from step 2. Otherwise, the best individual of the current population is the optimum parameter.

In this study, the GA parameters are selected as follows: Maximum number of iteration = 5000; Population size = 300; Crossover rate = 0.80; Mutation rate = 0.03.

### Error evaluation metrics of model accuracy

When evaluating the accuracy of model fitting, the statistical indices including RMSE, the coefficient of determination *R*, mean absolute error (MAE) and mean absolute percentage error (MAPE) are used to judge the goodness-of**-**fit of different models to wind speed and power data. Because RMSE indicates the root mean square error between the forecast values and observed values, and the coefficient of determination *R* quantifies the correlation between the predicted values and observed values. They are defined respectively by

20 $$ {\text{RMSE}} = \sqrt {\frac{1}{n}\sum\nolimits_{i = 1}^{n} {\left( {y_{i} - \hat{y}_{i} } \right)^{2} } } = \sqrt {\frac{{{\text{RSS}}}}{n}} $$

21 $$ R^{2} = 1 - \frac{{\sum\nolimits_{i = 1}^{n} {\left( {y_{i} - \hat{y}_{i} } \right)^{2} } }}{{\sum\nolimits_{i = 1}^{n} {\left( {y_{i} - y_{m} } \right)^{2} } }} $$

22 $$ {\text{MAE}} = \frac{1}{n}\sum\nolimits_{i = 1}^{n} {\left| {y_{i} - \hat{y}_{i} } \right|} $$

and

23 $$ {\text{MAPE}} = \frac{1}{n}\sum\nolimits_{i = 1}^{n} {\left| {\frac{{y_{i} - \hat{y}_{i} }}{{y_{i} }}} \right|} $$

where RSS is the residual sum of squares, *y*_*i*_ is the *i*th observed value, *y*_*m*_ is the mean value of all observations, and $\hat{y}_{i}$ is the *i*th estimated value, respectively. The high values of RMSE, MAE and MAPE indicate a poor fit, and the smaller these values are, the higher the model fitting accuracy is. Unlike RMSE, MAE and MAPE, a larger *R*^2^ value indicates that the proposed model fits the wind data well in all candidate models and has a higher fitting accuracy.

To avoid the overfitting problem of fitting model, the AIC and BIC are also used to select the best model in all candidate models. This is because that AIC criterion considers both model complexity and fitting accuracy, and the lower value of AIC indicates that the model is fitted well relatively, compared with AIC information criterion, BIC can avoid overfitting resulting by increasing the number of model parameters by introducing a penalty term for the extra number of parameters, similarly the model with the lowest value of BIC prefers as the best model, the corresponding expressions of AIC and BIC are given as^2,3^:

24 $$ {\text{AIC}} = - 2\max \ln L + 2q,{\text{ BIC}} = - 2\max \ln L + q\ln n $$

where* q* is the number of model parameters to be estimated, *n* is the number of all samples to be fitted, and maxln*L* is the maximum log-likelihood of the model.

However, in nonlinear regression applications, instead of using the maximum log-likelihood, the RSS is used as a reference^37,38^. In this case, the improved AIC and BIC are rewritten as follows:

25 $$ {\text{AIC}} = n\ln \left( {{{{\text{RSS}}} \mathord{\left/ {\vphantom {{{\text{RSS}}} n}} \right. \kern-0pt} n}} \right) + 2q,{\text{ BIC}} = n\ln \left( {{{{\text{RSS}}} \mathord{\left/ {\vphantom {{{\text{RSS}}} n}} \right. \kern-0pt} n}} \right) + q\ln n $$

Thus, the optimal model can be selected by comparing the calculating results of various types of power curves with four evaluation indices of RMSE, *R*^2^ and improved AIC and BIC.

## Wind speed modelling and wind power prediction

Using WPC model and wind speed model, the annual energy production (AEP) of wind turbines can be calculated^4^, the working status of wind turbine can also be monitored. Among them, the working status monitoring of wind turbines is mainly applied to the real-time monitoring and fault determination of wind turbines. This section focuses on the application of WPC in AEP and wind power prediction.

### Wind speed distribution

The two-parameter Weibull distribution is most commonly used in wind speed probability distribution modelling because of its simplicity and generality. The probability density function (pdf) of a two-parameter Weibull distribution is^39^:

26 $$ f(v) = \left( {{\beta \mathord{\left/ {\vphantom {\beta \eta }} \right. \kern-0pt} \eta }} \right)\left( {{v \mathord{\left/ {\vphantom {v \eta }} \right. \kern-0pt} \eta }} \right)^{\beta - 1} \exp \left[ { - \left( {{v \mathord{\left/ {\vphantom {v \eta }} \right. \kern-0pt} \eta }} \right)^{\beta } } \right] $$

where *β* is shape parameter, *η* is scale parameter, *β* and *η* > 0, and *v* is wind speed.

When the distribution of wind speed is bimodal or multimodal, mixture distributions have a good fitting accuracy than a single distribution. The mixture distributions are a linear combination of two or several single distributions, then based on Eq. (26), the pdf of a *M*-component Weibull mixture distributions is^3,40^:

27 $$ \begin{gathered} f(v) = \sum\nolimits_{i = 1}^{M} {w_{i} f(v;\eta_{i} ,\beta_{i} )} \hfill \\ \, = \sum\nolimits_{i = 1}^{M} {w_{i} \left( {{{\beta_{i} } \mathord{\left/ {\vphantom {{\beta_{i} } {\eta_{i} }}} \right. \kern-0pt} {\eta_{i} }}} \right)\left( {{v \mathord{\left/ {\vphantom {v {\eta_{i} }}} \right. \kern-0pt} {\eta_{i} }}} \right)^{{\beta_{i} - 1}} \exp \left[ { - \left( {{v \mathord{\left/ {\vphantom {v {\eta_{i} }}} \right. \kern-0pt} {\eta_{i} }}} \right)^{{\beta_{i} }} } \right]} \hfill \\ \end{gathered} $$

where *M* denotes the number of Weibull mixture distributions, *w*_*i*_ is the weight of each distribution, and the following relationship needs to be satisfied.

28 $$ 0 \le w_{i} \le 1, \, \sum\nolimits_{i = 1}^{M} {w_{i} = 1} $$

Therefore, there are more parameters need to be estimated in mixture distributions than single distribution. The maximum likelihood estimators of model parameters can be obtained by an EM algorithm^3^.

### Wind power prediction using AEP

AEP of a specific wind turbine is predicted based on the pdf of wind speed and WPC model, and can be given by^3^:

29 $$ {\text{AEP}} = N_{h} \int_{{\text{cut - in}}}^{{\text{cut - out}}} {P\left( v \right)} f\left( v \right){\text{d}}v $$

where* N*_*h*_ is the number of annual power generation time, calculated as 8760 h.

## Case study

SCADA data were collected in a period of one year (January 1, 2018 to December 31, 2018) from 1# wind farm Maling Mountain (34°31′ N and 118°44′ E), located in Jiangsu Province, China. Wind data used in this study consisted of the daily averaged 10-min wind speed and wind power output, and from 28 same types of wind turbines. The hub height is 85 m, the cut-in speed is 2 m/s, the cut-out speed is 18 m/s, the rated speed is 10 m/s, and the rated power is 1800 kW. In this case study, the wind speed range is 0 ~ 18 m/s, and the average wind speed is 5.62 m/s. The optimal wind speed model is fitted by a two-component Weibull mixture distribution, for more details of our previous work see Reference 3. The estimated parameters are given as follows: *w*_1_ = 0.8726, *β*_1_ = 2.5368, *η*_1_ = 4.8927; *w*_1_ = 0.1274, *β*_1_ = 6.1139, *η*_1_ = 4.5783. Therefore, the pdf of wind speed is

30 $$ \begin{gathered} f\left( v \right) = 3.9433 \times 10^{ - 2} v^{1.5368} \exp \left( { - 1.7814 \times 10^{ - 2} v^{2.5368} } \right) \hfill \\ \, + 7.1123 \times 10^{ - 5} v^{5.1139} \exp \left( { - 9.1310 \times 10^{ - 5} v^{6.1139} } \right) \hfill \\ \end{gathered} $$

Based on the analysis results of the bins method, a total of 36 fitting points of wind data including wind speed and wind power are taken. The wind data are shown in Table 1.

**Table 1:** Fitting points of wind speed-power data for 1# wind farm.

No	*v* (m/s)	*P* (kW)	No	*v* (m/s)	*P* (kW)	No	*v* (m/s)	*P* (kW)
1	0.126	− 3.151	13	6.234	838.259	25	12.245	1838.213
2	0.799	− 3.181	14	6.738	1039.989	26	12.715	1839.370
3	1.287	− 3.213	15	7.243	1229.457	27	13.226	1838.915
4	1.749	− 3.187	16	7.728	1411.141	28	13.743	1839.852
5	2.259	2.824	17	8.232	1540.810	29	14.300	1840.354
6	2.719	20.331	18	8.741	1681.272	30	14.696	1840.536
7	3.284	74.153	19	9.219	1783.571	31	15.232	1840.206
8	3.751	132.315	20	9.704	1818.255	32	15.649	1840.670
9	4.246	225.115	21	10.230	1822.494	33	16.029	1839.220
10	4.745	347.751	22	10.724	1821.436	34	16.803	1838.905
11	5.244	486.502	23	11.211	1838.905	35	17.049	1829.330
12	5.724	660.094	24	11.767	1840.394	36	17.885	1837.585

For data comparative analysis, and verification of the scope of application of the method proposed in this paper. Another group wind data from 2# wind farm Mishan Mountain (45°42′ N and 132°16′ E), located in Heilongjiang Province, China, are also collected. The hub height is 70 m, the cut-in speed is 3 m/s, the cut-out speed is 18 m/s, the rated speed is 10.5 m/s, and the rated power is 1500 kW. In this case study, the wind speed range is 0 ~ 18 m/s, and the average wind speed is 7.48 m/s. The wind speed-power data are shown in Table 2.

**Table 2:** Fitting points of wind speed-power data for 2# wind farm.

No	*v* (m/s)	*P* (kW)	No	*v* (m/s)	*P* (kW)	No	*v* (m/s)	*P* (kW)
1	0.127	0	13	6.242	427.592	25	12.223	1530.936
2	0.762	0	14	6.742	559.816	26	12.747	1529.599
3	1.264	0	15	7.240	693.596	27	13.177	1536.726
4	1.751	0	16	7.737	835.877	28	13.751	1522.060
5	2.251	− 0.085	17	8.232	1001.172	29	14.127	1539.429
6	2.740	− 0.068	18	8.733	1142.401	30	14.682	1539.903
7	3.315	32.359	19	9.231	1293.646	31	15.208	1538.153
8	3.786	68.850	20	9.735	1394.011	32	15.762	1502.114
9	4.251	117.135	21	10.234	1479.036	33	16.191	1531.601
10	4.751	176.413	22	10.740	1515.729	34	16.633	1518.205
11	5.249	239.551	23	11.218	1514.408	35	17.185	1504.880
12	5.745	321.004	24	11.730	1536.201	36	17.570	1541.745

## Results and discussion

At first, the 5–9th order polynomial models are all used to fit WPC for wind farms 1# and 2#, respectively, the model parameters are estimated directly by LSE method, six evaluation metrics to the different high-order polynomial models are calculated, the results are shown in Tables 3 and 4. From Table 3, it can be seen that with the increasing of the order, the value of *R*^2^ increases gradually, while the values of RMSE, AIC and BIC monotonically decreases, which indicates that the fitting accuracy of the WPC model is getting higher and higher. It is also found that the 8th order polynomial has the lowest values of MAE and MAPE. Therefore, the 8th and 9th order polynomials are often used to model WPC^5,10,41^.

**Table 3:** Modelling results of wind power curve with different high-order polynomial for 1# wind farm.

Coefficients and evaluation metrics	Orders of polynomials				
	*m* = 5	*m* = 6	*m* = 7	*m* = 8	*m* = 9
*a*_9_					− 8.656E−6
*a*_8_				1.044E−4	8.064E−4
*a*_7_			3.403E−4	− 7.191E−3	− 3.099E−2
*a*_6_		− 5.908E−3	− 0.0273	0.1942	0.6315
*a*_5_	2.022E−3	0.3211	0.8546	− 2.5604	− 7.2771
*a*_4_	0.1866	− 6.3262	− 12.9677	16.4820	46.7102
*a*_3_	− 9.6974	52.4844	95.5657	− 44.6123	− 155.4505
*a*_2_	127.0131	− 149.3618	− 286.3300	51.1578	261.4265
*a*_1_	− 345.8912	137.1217	311.6994	− 18.0634	− 181.9782
*a*_0_	165.3935	− 22.0516	− 69.6574	− 3.6291	21.7905
*R*^2^	0.9913	0.9983	0.9988	0.9996	**0.9997**
RMSE	71.7361	31.7569	27.0385	14.7986	**12.4454**
MAE	4.6822	1.3359	2.0420	**0.9416**	1.4298
MAPE	3.8758	0.5779	1.3617	**0.1374**	0.5561
AIC	319.6556	262.9838	253.4028	212.0065	**201.5374**
BIC	329.1567	274.0685	266.0710	226.2581	**217.3726**

The values that are in bold represent best values.

**Table 4:** Modelling results of wind power curve with different high-order polynomial for 2# wind farm.

Coefficients and evaluation metrics	Orders of polynomials				
	*m* = 5	*m* = 6	*m* = 7	*m* = 8	*m* = 9
*a*_9_					1.830E−5
*a*_8_				8.563E−5	− 1.372E−3
*a*_7_			− 4.652E−4	− 6.526E−3	4.201E−2
*a*_6_		− 3.509E−3	0.0253	0.2003	− 0.6759
*a*_5_	2.521E−2	0.2117	− 0.4935	− 3.1406	6.1487
*a*_4_	− 0.9586	− 4.7047	3.9265	26.3322	− 32.2001
*a*_3_	10.4515	45.6565	− 9.4102	− 114.1087	97.0289
*a*_2_	− 17.6341	− 171.6884	0.5650	248.1433	− 146.2617
*a*_1_	− 29.9454	235.1159	18.9316	− 219.0175	84.4388
*a*_0_	33.8203	− 66.8118	− 8.6984	38.6571	− 8.7383
*R*^2^	0.9949	0.9976	0.9986	0.9991	**0.9996**
RMSE	46.6738	31.7117	24.7158	19.0632	**12.2839**
MAE	39.8377	27.0293	20.5837	16.9589	**9.2905**
MAPE	15.2281	**4.0494**	5.4152	19.3005	5.9125
AIC	288.7091	262.8813	246.9360	230.2387	**200.5966**
BIC	298.2102	273.9660	259.6042	244.4904	**216.4318**

The values that are in bold represent best values.

Similar to 1# farm, from Table 4, it can be found that for 2# farm, 9th order polynomial still gives the best wind speed-power model, because except the value of MAPE, 9th order polynomial has the highest value of *R*^2^ and the lowest values of RMSE, MAE, AIC and BIC.

**Figure 4:**
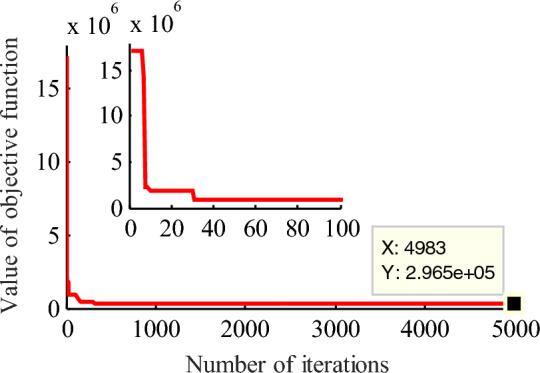
GA iteration results for five-parameter logistic function with 5000 generations for 1# wind farm. The partial enlarged detail is also given in the top left corner. Figure created using Matlab R2014a (8.3.0.532). (https://www.mathworks.com).

**Figure 5:**
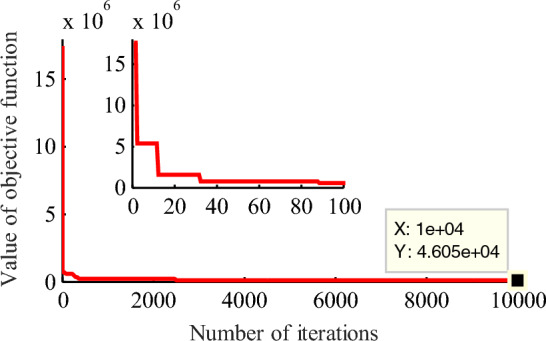
GA iteration results for five-parameter logistic function with 10,000 generations for 1# wind farm. The partial enlarged detail is also given in the top left corner. Figure created using Matlab R2014a (8.3.0.532). (https://www.mathworks.com).

For 1# wind farm, using GA, the initial value for 4PLF and 5PLF models are obtained as follows: ***θ***_4_ = [1839, − 5, 589, 1] and ***θ***_5_ = [1820, − 17, 10, 4, 5]. To analyze the effect of the number of iterations to the estimation results for the same model, a compassion for 5PLF with the number of iterations are 5000 and 10,000 is given, the results are shown in Figs. 4 and 5. It can be found that increasing the number of iterations, the final value of objective function is decreasing from 2.965 × 10^5^ to 4.605 × 10^4^, the estimation accuracy is slightly improved, but this improvement is limited and extremely time-consuming.

For 1# farm, based on the initial values obtained by GA, the parameter estimator for 4PLF and 5PLFs using the GLSE method are ***θ***_4_ = [1851, − 3.887, 345.3, 1.092] and ***θ***_5_ = [1832, − 13.9, 34.55, 4.016, 608.5], the evaluation indices are shown in Table 5. From Table 5, it can be found that the parameter estimation results given by GLSE method are more accurate. The value of RMSE for 4PLF is 26.4906 when model parameters are estimated by GA only, while the estimation value of RMSE obtained by GLSE method becomes 20.5201, which is reduced by 5.9705. The value of RMSE for 5PLF is also reduced from 35.7661 to 12.1018 after using GLSE, it is reduced by 23.6643. The value of *R*^2^ all increase after using GLSE method. It is also found that the 9th order polynomial has the lowest values of MAE and MAPE, they are 9.3156 and 0.3374, respectively. It is worth noting that if only GA is used to estimate model parameter, due to the phenomenon of prematurity in the selection procedure based on the fitness, although the number of iterations has reached 10,000 generations, the accurate results are still not achieved. Comparing the values of AIC and BIC for different WPC models in Tables 3 and 5, it can be determined that the optimal model is 5PLF (highlighted in bold in Table 5) with the lowest values of AIC and BIC, they are 189.5216 and 197.4392, which followed by the ninth-order polynomial, its values of AIC and BIC are 201.5374 and 217.3726. The values of AIC and BIC of the eighth-order polynomial are 212.0065 and 226.2581, and 4PLF are 225.5411 and 231.8751. Therefore, the eighth-order polynomial and 4PLF are ranked third and fourth, respectively.

**Table 5:** Logistic function fitting results with different methods for 1# wind farm.

Evaluation metrics	GA for 4PLF	GLSE for 4PLF	GA for 5PLF	GLSE for 5PLF
*R*^2^	0.9988	0.9993	0.9978	**0.9998**
RMSE	26.4906	20.5201	35.7661	**12.1018**
MAE	17.8797	15.9986	29.0502	**9.3156**
MAPE	0.3384	0.4641	0.4732	**0.3374**
AIC	243.9288	225.5411	267.5440	**189.5216**
BIC	250.2628	231.8751	275.4616	**197.4392**

The values that are in bold represent best values.

**Figure 6:**
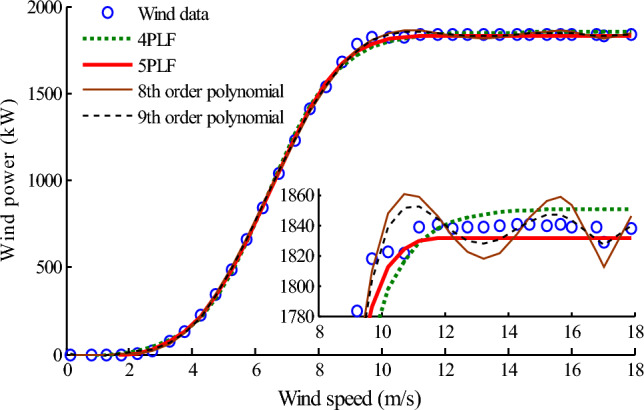
Comparison of the fitting results for 1# wind farm. Figure created using Matlab R2014a (8.3.0.532). (https://www.mathworks.com).

The fitting results of four model including the eighth-order and ninth-order polynomials, 4PLF and 5PLF of 1# farm are all shown in Fig. 6. The partial enlarged detail is also given in the bottom right corner. It can be found that the estimated results given by the high-order polynomial model fluctuate in the range of 40 kW and give an overfitting. Even if wind speed has already exceeded the rated wind speed, the estimation values of the high-order polynomial model still fluctuate with wind data points, and this phenomenon is more obvious when there are fewer wind data points. Compared with the high-order polynomial model, the estimated results of LF model obtained by GLSE method are more smooth and stable, and avoid this overfitting. This is because that the former has more model parameters than that of the latter. Hence, the LF can be preferred compared with high-order polynomial when the fitting accuracy is close. On the other hand, the 5PLF is better than the 4PLF, this conclusion is same as that of Villanueva and Feijoo^23^. In this study, the 4PLF model give an overestimate on wind power of about 10 kW at each data point after the rated speed. A possible explanation is that the 4PLF model assumes a symmetrical curve around the inflexion point and is not sufficient when the sigmoidal curve is not symmetrical around the inflexion point. However, there is a very high possibility that the variation trend of wind power around the inflexion point is not symmetrical. Fitting curves obtained by 4PLF, however, are point symmetric on the semi-log axis about its midpoint, which cannot accurately fit the power curves with asymmetric features^22^. The 5PLF model, which assumes an asymmetrical variation trend around the inflexion point, could be a better choice^42^.

For 2# wind farm, using GA, the initial value for 4PLF and 5PLF models are obtained as follows: ***θ***_4_ = [1722, 7, 93, 2] and ***θ***_5_ = [1530, 23, 15, 5, 23]. Based on the initial values obtained by GA, the parameter estimator for 4PLF and 5PLFs using the GLSE method are ***θ***_4_ = [1545, − 0.1184, 585.8, 1.163] and ***θ***_5_ = [1530, 20.03, 53.92, 4.621, 6420], the evaluation indices are shown in Table 6. It can be seen that except the value of MAPE, 5PLF model has the highest value of *R*^2^ and the lowest values of RMSE, MAE, AIC and BIC, which shows that 5PLF is superior to 4PLF. Comparing the values of AIC and BIC for different WPC models in Tables 4 and 6, it can be found that unlike 1# farm, for 2# farm, the optimal model is the ninth-order polynomial with the lowest values of AIC and BIC, they are 200.5966 and 216.4318, which followed by 5PLF, its values of AIC and BIC are 217.6608 and 225.5783. The values of AIC and BIC of the eighth-order polynomial are 230.2387 and 244.4904, and 4PLF are 240.4844 and 246.8185. Therefore, the eighth-order polynomial and 4PLF are ranked third and fourth, respectively.

**Table 6:** Logistic function fitting results with different methods for 2# wind farm.

Evaluation metrics	GA for 4PLF	GLSE for 4PLF	GA for 5PLF	GLSE for 5PLF
*R*^2^	0.9280	0.9985	0.9987	**0.9992**
RMSE	174.8807	25.2531	23.5812	**17.8888**
MAE	153.4549	19.9782	19.5477	**15.3001**
MAPE	156.1869	**19.0507**	23.3403	22.8281
AIC	379.8155	240.4844	237.5523	**217.6608**
BIC	386.1496	246.8185	245.4699	**225.5783**

The values that are in bold represent best values.

**Figure 7:**
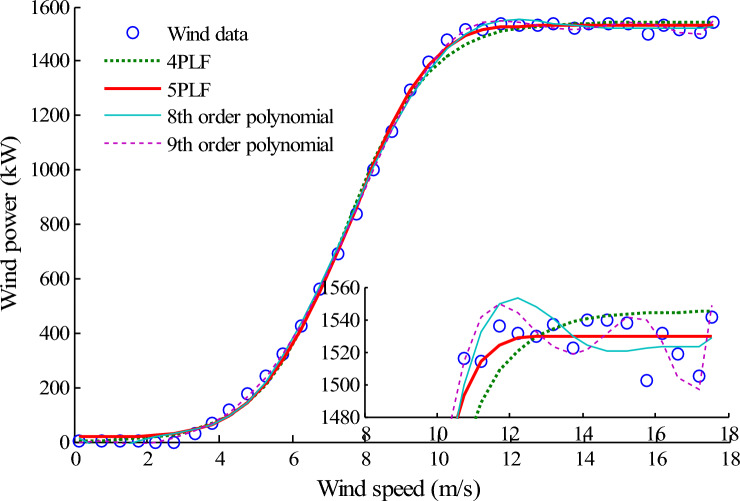
Comparison of the fitting results for 2# wind farm. Figure created using Matlab R2014a (8.3.0.532). (https://www.mathworks.com).

The fitting results of four model including the eighth-order and ninth-order polynomials, 4PLF and 5PLF of 2# farm are all shown in Fig. 7. The partial enlarged detail is also given in the bottom right corner. Based on Fig. 7, the same conclusion can be drawn as obtained from 1# farm, the estimated results given by the high-order polynomial model fluctuate in a range and give an overfitting. However, the estimated results of LF model obtained by GLSE method are more smooth and stable, and avoid this overfitting. In this case, the LF model is recommended when the modelling accuracy is close.

Consequently, the 5PLF model give a better fitting results than 4PLF model for 1# farm, and the wind power model is given by

31 $$ P(v) = 1832 - \frac{1845.9}{{\left[ {1 + \left( {{v \mathord{\left/ {\vphantom {v {34.55}}} \right. \kern-0pt} {34.55}}} \right)^{4.016} } \right]^{608.5} }} $$

Using Eq. (31), the estimated cut-in speed and rated speed can be obtained as 2.07 m/s and 9.93 m/s, respectively. Compared with the actual cut-in speed 2 m/s and rated speed 10 m/s, the maximum relative error is 0.035. Finally, using Eqs. (29), (30) and (31), the predicting results of AEP for 1# farm are shown in Table 7.

**Table 7:** Calculation results of AEP. For comparison, one-year wind power before and after cleaning abnormal data, named here the actual and available wind power, are also given.

Wind power	AEP (GWh)
Predicted wind power	3.2360
Actual wind power	3.1725
Available wind power	2.5973

To verify the calculating results, based on one-year period wind power data from 1# farm, the actual total wind power is 3.1725 GWh. Compared with this value, the AEP estimation result of 3.2360 GWh is close to the actual AEP, the relative error is only 2.00%. Therefore, the correctness of the method proposed in this paper is validated. On the other hand, the utilization rate of wind energy is 81.87%, it means that wind energy resources are not used efficiently, the possible reasons are caused by the failures of wind turbines and measuring instrument, wind abandonment or ration electricity, environmental conditions, and maintenance, etc.^5,21^.

**Figure 8:**
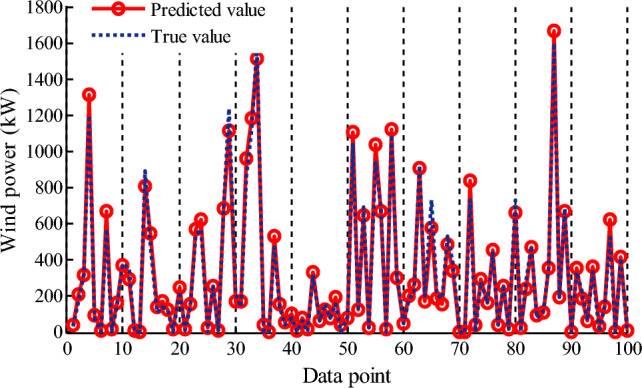
Wind power prediction with five-parameter logistic model for 1# farm. Figure created using Matlab R2014a (8.3.0.532). (https://www.mathworks.com).

To predict wind power, wind speed prediction is required after obtaining the power curve for a specific turbine. In this paper, a data set of real wind speed from 1# farm is used directly to verify the accuracy of power curve. Therefore, 100 wind speed data are randomly selected in real wind data, and after substituting the wind speed into the 5PLF WPC model, the predicting value of output power at that wind speed point can be obtained. The actual power values and the predicted values are shown in Fig. 8. It can be found that the actual power values at different wind speed data points are very close to the predicted power values, and the total actual output power of these 100 data points is 40,042 kW, and the predicted output power is 38,403 kW with a low relative error of 4.27%, indicating the WPC model has a high prediction accuracy. It also shows that proposed method of the power prediction based on wind turbine power curve is feasible.

## Conclusion

A GLSE approach, which combined GE with LSE to model wind turbine power curve and predict wind turbine AEP, are proposed in this study, the problem of selecting initial value of model parameter estimation for LF is solved, and the effectiveness and correctness are validated by the two-group different field wind data. The main conclusions are drawn as follows:

(i) The polynomial and LF models in modelling WPC were compared by six evaluation indices including RMSE, *R*^2^, MAE, MAPE, improved AIC and BIC, and it was found that 5PLF model outperforms 4PLF model, and both nine-order polynomial and 5PLF have a higher fitting accuracy. It is also found that the power values estimated by the high-order polynomial are still fluctuate even if wind speed far exceeds the rated wind speed. The LF model best describes the trend of wind power with wind speed and can be adopted to fit the relationship between wind speed and wind power. Therefore, the LF model is recommended when the modelling accuracy is close.

(ii) Although the LF is more suitable for the modelling of WPC than high-order polynomial, the LF requires an initial value when estimating the model parameters, and if the initial value is not selected appropriately, it will fall into a local optimum. Therefore, other algorithms are needed to be combined to search for a reasonable initial value. If an optimization algorithm is only used to estimate model parameters, it is time-consuming to convergence, combined GA with LSE, which not only can effectively estimate model parameter, but also significantly improve the estimation accuracy.

(iii) Based on the models of wind speed and power curve, APE can be obtained. It also proves that combined with wind speed estimation, it is possible to achieve an accurate wind power prediction using WPC and provide a reliable support for wind power grid connection and dispatching.

## Data Availability

All data generated or analysed during this study are included in this published article.
